# Dining with liberals and conservatives: The social underpinnings of food neophobia

**DOI:** 10.1371/journal.pone.0262676

**Published:** 2022-01-27

**Authors:** Margherita Guidetti, Luciana Carraro, Nicoletta Cavazza

**Affiliations:** 1 Dipartimento di Comunicazione ed Economia, Università di Modena e Reggio Emilia, Reggio Emilia, Italy; 2 Dipartimento di Psicologia dello Sviluppo e della Socializzazione, Università di Padova, Padova, Italy; Tohoku University: Tohoku Daigaku, JAPAN

## Abstract

Although food and politics seem to be distant domains, socio-political ideology and food neophobia (i.e., reluctance to eat unfamiliar food) may be related. Conservatives’ high threat sensitivity and the inherently threatening nature of novel foods (the existential explanation), along with conservatives’ negative attitudes toward minority outgroups (e.g., foreigners) and the role of the latter in introducing novel foods to a culture (the social explanation), led us to expect that socio-political ideology would predict food neophobia over and above their common roots. Across two correlational and two experimental studies (*N* = 627), socio-political ideology emerged as a strong predictor of food neophobia. In addition, the findings did not support the existential explanation, while confirming the social explanation of the ideology–food neophobia link: Conservatives seem more neophobic than liberals not because of their higher threat sensitivity but rather because they hold more negative attitudes toward foreigners who are associated with those foods.

## Introduction

James and Harry are dining out, and both notice the exotic tempeh with pineapple and papaya on the menu. However, they have different reactions: While James would not taste it even if they paid him, Harry is very curious about this novel and unusual dish and dares to order it. James and Harry can likely be placed on opposite ends of the food neophobia dimension, namely the reluctance to eat unfamiliar foods. This “fear of the new” is a universal predisposition among humans and, more generally, omnivores [1,2]. From an evolutionary perspective, each novel food represents both an opportunity and a risk: the opportunity to expand one’s nourishment source set and the risk of ingesting an item that is dangerous to one’s health or even life threatening. According to Paul Rozin [3], food neophobia arises from this “omnivore dilemma.”

Although food neophobia serves a protective function in potentially dangerous environments, it can be problematic in contemporary societies characterized by food safety because it limits consumption variety. In fact, food neophobia adversely affects both adults’ and children’s eating preferences and diet quality [e.g., 4–6], is positively correlated with women’s BMI [7], and is associated with a higher incidence of chronic diseases [8]. In addition, the current scenario of global warming urges all consumers to change their habits: More plant-based diets would be particularly effective in reducing humans’ negative impact on the environment [e.g., 9]. High levels of food neophobia, representing an obstacle to fruit and vegetable consumption [e.g., 7] and acceptance of meat substitutes [e.g., 10], hinder individuals’ health as well as environmental protection. Therefore, identifying the antecedents of food neophobia and effective intervention strategies is critical. Many studies have been devoted to this purpose, focusing on socio-demographic characteristics, personality traits and emotions (for a review, see [11]). However, we believe that, to deeply understand this phenomenon at present, we must examine not only its existential but also its social meaning. Why, in a safe context, do people remain averse to novel food?

Rozin and Fallon [12,13] proposed a taxonomy of motivations for food rejection in general: Two out of three such motivations can be useful in answering our question. According to the authors, food can be rejected on the basis of disliked sensory characteristics, such as taste, texture, or smell (distaste); fear of negative effects of ingestion (danger); or the idea of what a food is or where it comes from (disgust). The distaste category is not helpful in explaining the specific phenomenon of food neophobia mainly because it implies tasting the food, whereas “the concept of food neophobia only extends to the point where the individual picks up the food and places it in his mouth” [14]. In other words, neophobic individuals refuse to taste novel food and thus cannot feel distaste after trying it. Danger and disgust, in contrast, seem more useful in relation to the specific rejection of new foods that are potentially dangerous by nature and typically come from outside one’s own culture or subculture, being introduced to one’s environment by social outgroups, such as immigrants or vegans.

Scholars have not yet investigated these two specific motivations in relation to food neophobia. In our effort to carry out such work, we built upon two apparently unrelated literatures, the first dealing with food neophobia and the second with political ideology. The latter concept is not only a matter of politics but rather represents a lens through which people view reality at large and forge their general mindsets. The several differences observed between liberals and conservatives fit into the model of political ideology as motivated social cognition [15–17], according to which ideology meets individuals’ epistemic, existential, and relational needs for certainty, security, and solidarity. In other words, a dispositional or situational heightened motivation to reduce uncertainty, threat, or isolation drives individuals to prefer stability over change and to accept inequalities, which are the two core aspects of conservative ideology.

Left–right differences in personality, cognitive style, motivational concerns, and personal values are also manifested in everyday life, as Carney et al. [18] have shown looking at nonverbal behavior in the context of social interactions and characteristics of living and working spaces. In the food domain, it has been shown that people endorsing conservative values and beliefs consume more meat, identify themselves as meat eaters, are less likely to be vegetarian [19–23], and hold more negative attitudes toward vegetarians [24,25] compared with people holding liberal views.

Another consumer behavior linked to political ideology could be food neophobia. Indeed, danger and disgust as reasons for rejecting novel foods should be particularly strong for conservatives: first, because they are more sensitive than liberals to threat (for a review, see [15]); and second, because novel foods usually enter a culture through social minorities, such as immigrants and vegans, and conservatives tend to hold more negative attitudes than liberals toward social minorities and outgroups [e.g., 24–26].

The two investigated concepts also share some predictors and associated factors. First, both conservative ideology [e.g., 27] and food neophobia [e.g., 28] are positively associated with disgust sensitivity. In addition, individuals’ conservatism increases with death anxiety and system threat (for a meta-analysis, see [29]); similarly, the food neophobia rises with situational fear and trait anxiety [e.g., 30,31]. Food neophobia is also correlated with general neophobia [e.g., 31], and we can suppose the same for conservatism, as resistance to change is one of its crucial determinants [16,17]. The two concepts also rely on the same personality trait, as highly neophobic individuals [7,32]—like conservative and authoritarian ones [33–39]—score low on the openness to experience dimension. Finally, we know that more educated people are also more liberal [40] and less neophobic [e.g., 5].

### The present research

The high threat sensitivity of individuals embracing conservative views [15] and the inherently threatening nature of novel food (the existential explanation), along with conservatives’ negative attitudes toward minority outgroups such as immigrants and vegans [e.g., 24–26] and the role of such groups in introducing novel foods to a culture (the social explanation), led us to anticipate that socio-political ideology would reinforce food neophobia over and above their common roots. We chose to focus on conservative versus liberal views on social issues (without considering economic issues) because most research to date has assessed or found associations between this type of ideological attitude and sociopsychological characteristics (e.g., [41]), although more recent work suggests that social and economic ideology are actually intertwined ([42]).

To test this prediction, we conducted four studies. Study 1 (correlational) aimed to explore whether socio-political ideology predicted food neophobia after controlling for common antecedents. In Study 2 (correlational), we tested whether the relation between ideology and food neophobia was moderated by both the threatening nature of novel foods and their association with foreigner outgroups. In Studies 3 and 4 (experimental), we manipulated the two aspects of conservatism that should promote food neophobia (namely, threat sensitivity and attitudes toward minority outgroups), anticipating that experimentally reducing these differences between conservatives and liberals would also eliminate or diminish their differences in terms of food neophobia.

For each study, we reported all measures and conditions as well as any data exclusions. The studies were conducted in accordance with the Declaration of Helsinki and have been approved by one of the authors’ local ethics committees. Supplemental materials (questionnaires, stimuli, and manipulations), data, and the syntax for all analyses are available at https://osf.io/9bkva/?view_only=3b555dd06fff4139ad0db421f58d57f2.

To increase generalizability, we sought to recruit heterogeneous adult samples (only Study 2 involved exclusively students as participants) and using different measures of the dependent variables. In each study, the sample size exceeded that revealed by a priori power analyses as sufficient to detect a medium-sized effect with at least .85 power.

## Study 1: Ideology and food neophobia

### Method

#### Participants and procedure

A total of 182 Italian participants (79.4% female) aged between 18 and 73 years (*M* = 39.96, *SD* = 10.89) were recruited through snowball sampling on Facebook. They completed an online questionnaire including sociodemographic data (age, gender, and education) and the measures described below. Five participants were dropped from the analysis because of a large Cook’s distance [43]. The final sample consisted of 140 women and 37 men. Their mean age was 39.89 (*SD* = 10.82); 36.2% had a high-school education, and 54.8% had a university-level degree. A sensitivity analysis conducted with G*Power [44] assuming an α of 0.05 and a power of 0.95 showed that our sample was sufficient to detect small effects of *f*^2^ = .06 for a linear multiple regression with six predictors.

#### Measures

General neophobia was assessed using the 8-item General Neophobia Scale (GNS; [31]; α = .80). Food neophobia was assessed using the 10-item Food Neophobia Scale (FNS; [31]) and computed as the Revised Food Neophobia Scale (FNS-R; [45]; α = .83). Participants answered on a 5-point scale ranging from 1 = *very descriptive of me* to 5 = *not at all descriptive of me*. “I feel uneasy in unfamiliar surroundings” is a sample item from the GNS, and “I don’t trust new foods” is a sample item from the FNS-R.

Next, participants took a willingness to taste novel food (WTNF) test which presented 10 pictures of novel food items (dragon fruit, granadilla, straw mushrooms, kiwano, dried persimmon, mangosteen, watermelon seeds, rambutan, tamarillo, and lotus nuts). For each food, participants were asked to indicate whether they knew it and whether they would taste it. The knowledge responses were given on the same 6-point scale used in Reverdy et al. [46], i.e., 1 (rescaled 0) = *Yes*, *I know it and I eat it often*; 2 (rescaled 0) = *Yes*, *I know it and I sometimes eat it*; 3 (rescaled .25) = *Yes*, *I know it*, *but I never eat it*; 4 (rescaled .50) = *No*, *I do not know it*, *but it strongly resembles a food that I know*; 5 (rescaled .75) = *No*, *I do not know it*, *but it slightly resembles a food that I know*; and 6 (rescaled 1) = *No*, *I do not know it and it does not resemble any food that I know*. Willingness-to-taste responses were given on a 4-point scale where 1 (rescaled 0) = *definitely no*; 2 (rescaled .333) = *probably no*; 3 (rescaled .666) = *probably yes*; and 4 (rescaled 1) = *definitely yes*. Drawing on [46], we computed WTNF score as the proportion of unknown foods that each participant was willing to taste to the total number of unknown foods. More specifically, we rescaled the knowledge and willingness responses as reported above to transform the willingness responses into probability levels and to ensure that both ratings were on the same 0–1 scale so that we could multiply them. Thereafter, we weighted the probability of tasting each food based on level of knowledge. For example, if a person was likely to taste (.666) a food that is unknown but very similar to a known food (.50), the correspondent score would be .333, whereas both the known food and the not-tasted food scored 0. We then divided the sum of these products by the sum of the knowledge ratings to make the final scores comparable across different levels of knowledge. The final WTNF score, measuring behavioral neophilia, ranged from 0 to 1.

Socio-political ideology was assessed by asking participants to report their level of agreement (1 = *not at all*, 5 = *very much*) with eight statements about different topics: immigration, abortion, medically assisted procreation, same-sex marriage, adoption by same-sex couples, soft drugs, use of embryonic stem cells, and euthanasia. Half of the items were conservatism oriented and the other half were liberalism oriented. This measure has been used in previous studies [e.g., 47,48]. After appropriate rescaling of the liberal items by assigning higher values to conservative opinions, we computed a mean score for each participant (α = .76).

As a measure of death anxiety, participants completed the 5-item Externally Generated Death Anxiety and the 5-item Meaning and Acceptance of Death sub-scales of the Death Anxiety Inventory [49]. Respondents were asked to indicate how well each item described them on the same 5-point scale used for the neophobia measures. We computed a total mean score for each participant (α = .86).

Openness to experience was assessed using the 7-point semantic differential items of the Italian version of the Big Five Observer [50], which measures this personality trait using eight pairs of bipolar adjectives (e.g., original vs. traditional). After appropriate rescaling, we computed a mean score for each participant (α = .70).

Disgust sensitivity was evaluated using the Disgust Scale Revised (DS-R; [51], modified by [52,53]). In the first part of the scale, respondents indicated how well each of 13 items described them on the same 5-point scale used for the neophobia and death anxiety measures (e.g., “It would bother me to see a rat run across my path in a park”). In the second part, respondents rated how disgusting each of 12 situations would be for them, on a 5-point scale ranging from 1 = *not disgusting at all* to 5 = *extremely disgusting* (e.g., “While you are walking through a tunnel under a railroad track, you smell urine”). We computed a mean score for the total scale (α = .73). Disgust sensitivity as a trait must not be confused with disgust as an ideational motivation for food rejection [12,13]. In our reasoning, the latter might be a reason why conservatives should be more neophobic than liberals, whereas the former is a common antecedent of both ideology and food neophobia.

Finally, participants were asked to indicate their position on a 10-point left–right political self-placement continuum and responded to three questions on diet and religiosity, which we did not use in the present study.

### Results and discussion

As shown in Table 1, both socio-political ideology and left–right self-placement were positively correlated with trait food neophobia (as measured by the FNS-R) and negatively correlated with WTNF score, which is a proxy of neophilic behavior. As expected, both the measures of ideology and those of food neophobia were also significantly correlated with nearly all control variables. Age and gender did not affect the ideology or neophobia measures.

**Table 1:** Descriptives and correlations for Study 1 measures.

		*M*	*SD*	2	3	4	5	6	7	8	9
1.	Socio-political ideology	2.16	.74	.48***	.32***	-.24**	.25**	.15°	-.26**	.33***	-.24**
2.	Left-right self-placement	3.92	1.97	-	.26**	-.19*	.12	-.02	-.29***	.20**	-.21**
3.	Food neophobia—FNS-R	2.03	.91		-	-.48***	.23**	.12	-.25**	.23**	-.18*
4.	Food neophilia—WTNF	.69	.18			-	-.21**	-.17*	.11	-.24**	.05
5.	General neophobia	2.20	.69				-	.28***	-.29***	.36***	-.18*
6.	Death anxiety	2.51	.89					-	-.05	.48***	-.03
7.	BFO Openness to experience	5.15	.71						-	-.21**	.15
8.	Disgust sensitivity	2.25	.41							-	-.31***
9.	Education (years)	15.33	3.17								-

*** *p* < .001

** *p* < .01

* *p* < 05

° *p* < .10.

In order to determine that the association between ideology and food neophobia was not spurious due to common covariates—such as general neophobia, death anxiety, disgust sensitivity, openness to experience, or educational level—we ran two linear regressions on participants’ FNS-R scores and two on their WTNF scores, entering either socio-political ideology or left–right self-placement as predictors as well as the control variables (Table 2). As expected, both food neophobia measures were significantly predicted by both measures of ideology, even after controlling for the covariates: The more conservative participants were, the more neophobic they were and the less willing they were to taste novel food. Interestingly, despite significant correlations among most measures (Table 1), only the ideology measures retained significance when all predictors of food neophobia were entered simultaneously into the regressions.

**Table 2:** Results of linear regression on participants’ food neophobia (both FNS-R and WTNF scores) predicted by socio-political ideology and left-right self-placement, as well as common antecedents (Study 1).

	FNS-R				WTNF			
	β	*t*	*p*	95% CI	β	*T*	*p*	95% CI
(Constant)		2.91	.004	.73, 3.83		6.16	< .001	.67, 1.31
Openness to exp.	-.14	-1.87	.063	-.38, .01	.01	.12	.906	-.04, .04
Disgust sensitivity	.05	.58	.560	-.28, .52	-.13	-1.41	.162	-.14, .02
Death anxiety	.03	.34	.732	-.14, .20	-.06	-.67	.503	-.05, .02
General neophobia	.09	1.14	.256	-.09, .33	-.12	-1.47	.145	-.08, .01
Education (years)	-.08	-1.08	.283	-.07, .02	-.06	-.70	.483	-.01, .01
Ideology	**.22**	**2.85**	**.005**	**.08, .46**	**-.17**	**-2.10**	**.037**	**-.08, .003**
	*R*^*2*^ = .16, *F*(6,165) = 5.32, *p* < .001, *f*^2^ = .19				*R*^*2*^ = .11, *F*(6,165) = 3.31, *p* = .004, *f*^2^ = .12			
	Β	*t*	*p*	95% CI	Β	*T*	*p*	95% CI
(Constant)		3.02	.003	.84, 4.04		6.03	< .001	.67, 1.32
Openness to exp.	-.14	-1.73	.086	-.38, .03	-.02	-.21	.832	-.05, .04
Disgust sensitivity	.07	.81	.422	-.24, .56	-.13	-1.37	.172	-.14, .03
Death anxiety	.02	.25	.806	-.15, .20	-.07	-.78	.434	-.05, .02
General neophobia	.11	1.39	.166	-.06, .36	-.15	-1.82	.071	-.08, .00
Education (years)	-.09	-1.16	.249	-.07, .02	-.04	-.45	.656	-.01, .01
Self-placement	**.18**	**2.25**	**.026**	**.01, .15**	**-.16**	**-1.97**	**.050**	**-.03, .00**	
	*R*^*2*^ = .14, *F*(6,162) = 4.52, *p* < .001, *f*^2^ = .17				*R*^*2*^ = .10, *F*(6,162) = 3.04, *p* = .008, *f*^2^ = .11				

The present findings confirm the ideology–food neophobia link, suggesting that it is not a spurious consequence of their common antecedents. Therefore, we propose that it is a direct causal relation from conservatism to neophobia based on conservatives’ high threat sensitivity (existential explanation) and intergroup attitudes (social explanation). Studies 2 to 4 aimed to test these hypotheses. Given that the associations between conservatism and liberalism and the considered individual differences (both food neophobia and other psychosocial measures) were stronger for socio-political ideology than for left–right self-placement, we focused on the former in the subsequent studies.

## Study 2: A correlational test of the existential and social explanations

Study 2 sought to test and compare the existential and social explanations of the link between socio-political conservatism and food neophobia. According to the existential explanation, as conservatives are more sensitive to threat than liberals [15], the threatening nature of novel foods should make those foods more unacceptable to the former than to the latter. In addition, according to the social explanation, as conservatives generally hold more negative attitudes than liberals toward foreigner outgroups such as immigrants [e.g., 26], the association between novel foods and those outgroups should translate into food neophobia for the former but not the latter. First, we explored whether participants generally associated novel foods with both threat and outgroups. As individuals lack previous experience with novel food, both associations are likely independent from their awareness [54,55]. For this reason, we decided to assess them using implicit measurements; This should also prevent social desirability bias and ensure more variance. Second, we reasoned that these associations, which are generally held by most people, may be boundary conditions for the ideology–food neophobia link to emerge. Indeed, if this link depends on conservatives’ high threat sensitivity, they should be more neophobic than liberals only (or particularly) when associating novel foods with threat. Similarly, if conservatives are more neophobic than liberals because of their negative attitudes toward outgroups, this difference should appear only (or particularly) when novel foods are associated with those outgroups.

Therefore, we hypothesized a model whereby conservatism would increase food neophobia, moderated by both the threatening nature of novel food and its association with foreigner outgroups. Since we have no reason to expect that conservative individuals are more likely than liberals to associate novel foods with either social outgroups or threat (i.e., ideology should not affect the associations of novel food with either outgroups or threat), we did not anticipate a mediation model. Indeed, in a previous pilot study (not reported here for reasons of brevity), we used an Implicit Association Test (IAT [56]) to test whether people hold an implicit association between novel food and foreigners and whether this association varies as a function of foreigners’ status and participants’ ideology. Before conducting this study, we ran a pre-test (*N* = 41) to select the low- versus high-status names to be used as stimuli. Results showed that participants (*N* = 191) did associate novel food with foreigners’ names, regardless of foreigners’ status or participants’ ideology.

### Method

#### Participants and procedure

A total of 140 first-year university students (82% female) took part in this study. Participants came to the laboratory and performed two Brief Implicit Association Tests (BIATs [57]), then filled out two questionnaires interspersed by a filler task (counting and indicating the number of syllables of 10 words appearing on the computer screen) intended to separate independent and dependent variables and thus reduce common method variance bias. Nine participants were dropped from the analysis because of a large Cook’s distance [43]. The final sample consisted of 106 women and 25 men. Their mean age was 19.53 years (*SD* = 1.57). A sensitivity analysis conducted with G*Power [44], assuming an α of 0.05 and a power of 0.95, showed that our sample was sufficient to detect small effects of *f*^2^ = .08 for a linear multiple regression with nine predictors.

#### Measures

**Fig 1:**
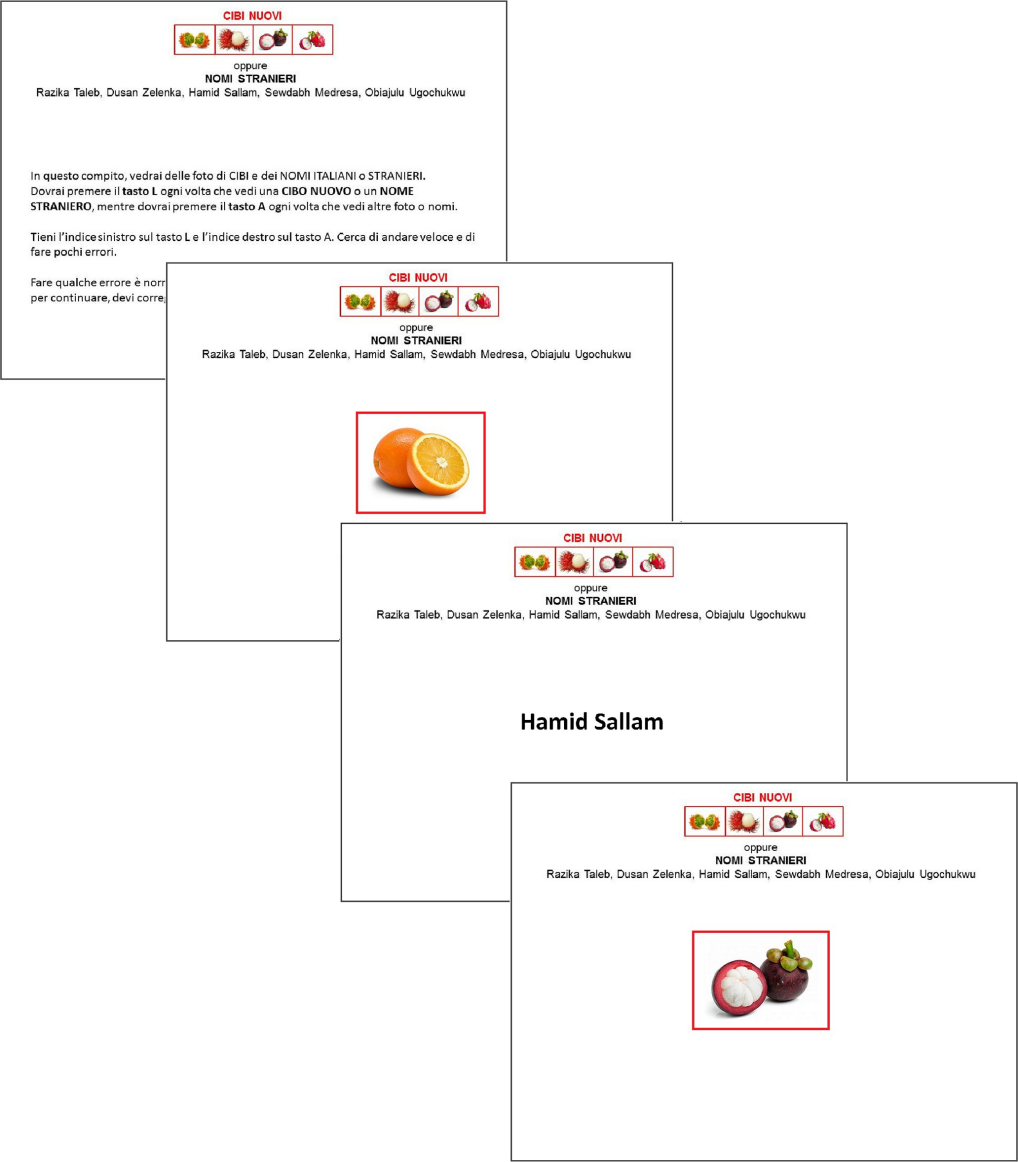
Examples of some BIAT trials. In this block, participants were asked to categorize stimuli by pressing the L key for both novel food pictures and foreigner names and the A key for all other stimuli.

The implicit associations between novel food and foreigner outgroups and between novel food and threat were assessed using two BIATs [57]. The BIAT is a shorter version of the IAT [56] that consists of two blocks of combined trials wherein participants are asked to focus on only one matched pair of categories at a time. Each block included 24 trials (for an example, see Fig 1). For both BIATs, stimuli for the two food categories “Familiar food” and “Novel food” were, respectively, four pictures of familiar fruits and four pictures of exotic fruits that had already been used in Study 1 and proven to be perceived as unfamiliar (all means significantly greater than 4 = *No*, *I do not know it*, *but it strongly resembles a food that I know*). For the food–outgroup BIAT, the focal category was “foreigner names,” which included four names perceived as belonging to low-status persons (as emerged in the pre-test to the pilot study mentioned above) who are more likely to be unwelcome to conservatives [58,59]. The non-focal category included four “Italian names” selected as common names typical of different Italian regions. For the food–threat BIAT, the focal category was “threat,” and participants were asked to recognize four threatening words drawn from previous research [60,61]: murderer, violence, bomb, and gun. The non-focal category included neutral words of the same length. The order of the two BIATs and the order of the blocks were both counterbalanced across participants. For each task, we computed a BIAT score as indicated by Nosek et al. [62] so that positive values indicated an association between novel food and foreigner names (for the food–outgroup BIAT) and between novel food and threat (for the food–threat BIAT).

The first questionnaire included socio-demographic information (age and gender), socio-political ideology (α = .65), and covariates, mostly assessed using the same scales as in Study 1: general neophobia (α = .80), openness to experience (α = .74), sensation seeking, and disgust sensitivity (α = .73). Openness to experience was measured using four items (already used in previous studies [63]) of a short 20-item version (five response categories) of the Italian Big Five Questionnaire [64]. In this study, we also assessed sensation seeking—a known correlate of food neophobia [31,32,65]—through the 12-item Italian version [66] of the Sensation Seeking Scale, Form V (SSS-V; [67]). As this scale is based on a dichotomous forced choice, we computed a total score as the sum of the items (range: 0–12) after properly rescaling responses so that the sensation-seeking option was coded as 1 and the opposite option was coded as 0. For explorative purposes, participants also completed a values measure that is not relevant to the present analyses.

The second questionnaire included the dependent variables as assessed in Study 1 (i.e., the FNS-R; [45]; α = .76) and the WTNF test. With regard to the WTNF, the structure, response options, and scoring of the measure were the same as in Study 1, but the 10 pictures of novel food items were different. They included the following dishes from various foreign countries rather than exotic fruits: baba ghanoush, dolmades, dorayaki, fabada, manti, pakchoy, souskai de platano, pork and ananas tacos, tajine of seabass in chermoula, and tavuk göğsü.

### Results and discussion

Table 3 displays the descriptive statistics and correlations for all measures. Ideology score was correlated with all covariates and food neophobia measures, which in turn correlated with most covariates. Participants’ age and gender affected neither the independent nor the dependent variables.

**Table 3:** Descriptives and correlations for Study 2 measures.

		*M*	*DS*	2	3	4	5	6	7	8	9
1.	Ideology	2.10	.57	.39***	-.37***	-.03	-.02	.19*	-.19*	.17*	-.37***
2.	Food neophobia FNS-R	2.50	.75	-	-.65***	.20*	.06	.30**	-.26**	.16	-.27**
3.	Food neophobia WTNF	.71	.17		-	-.08	-.02	-.14	.20*	-.18*	.29**
4.	Novel food-Outgroup BIAT	.73	.45			-	.29**	.11	.14	-.01	-.03
5.	Novel food-Threat BIAT	.46	.57				-	.01	.01	.01	.02
6.	General neophobia	2.35	.63					-	-.10	.44***	-.46***
7.	Openness to experience	3.65	.80						-	-.28**	.20*
8.	Disgust sensitivity	1.23	.40							-	-.45***
9.	Sensation seeking	7.60	2.72								-

*** *p* < .001

** *p* < .01

* *p* < 05.

Two one-sample *t*-tests showed that both BIAT scores were significantly greater than 0, confirming that participants tended to associate novel food with foreigner outgroups, *t*(130) = 18.53, *p* < .001, *d* = 1.61, 95% CI [.65, .81], as well as to threatening stimuli, *t*(130) = 9.14, *p* < .001, *d* = .80, 95% CI [.36, .56]. A paired-sample *t*-test showed that the first association was significantly stronger than the latter, *t*(130) = 4.97, p < .001, *d* = .43, 95% CI [.16, .38]. As shown in Table 3, neither BIAT score correlated with the ideology measure.

**Fig 2:**
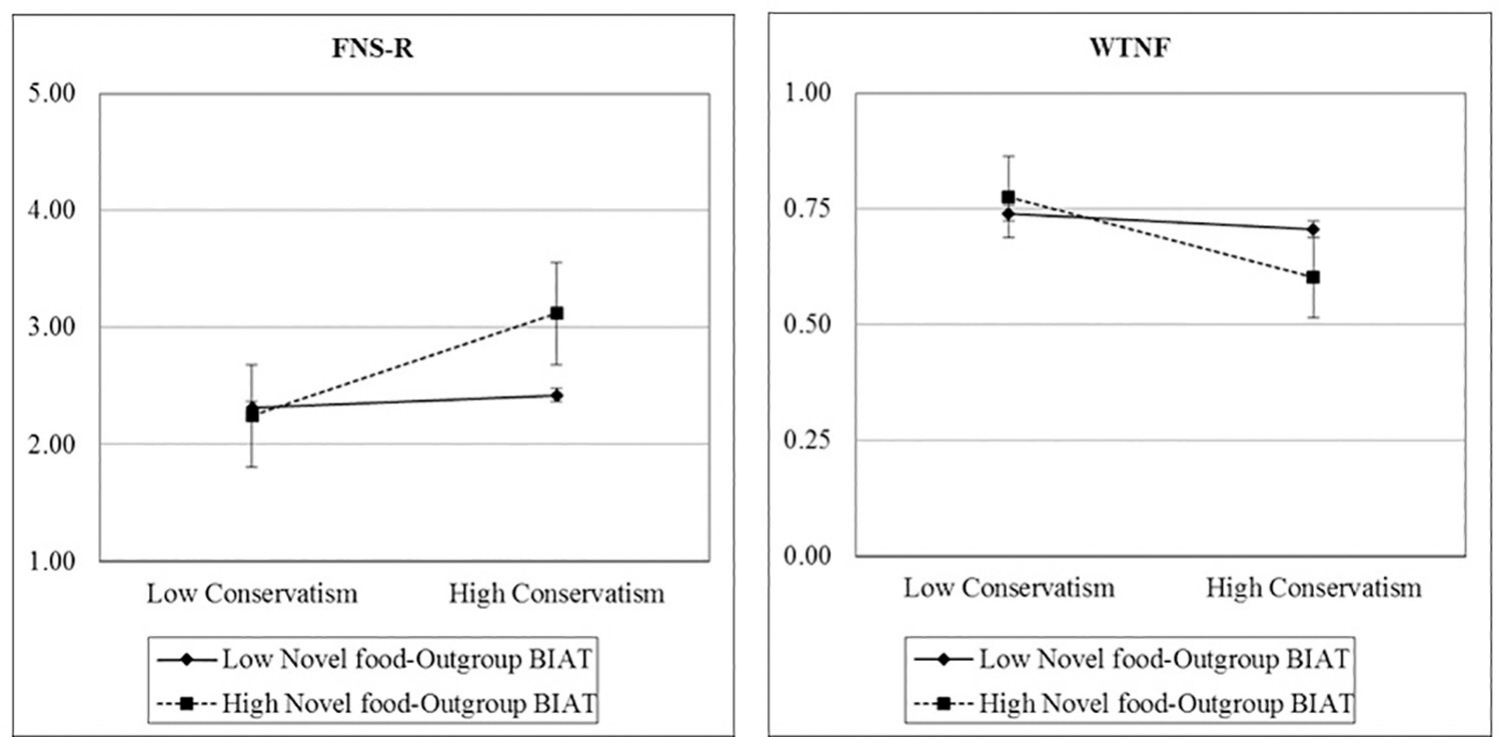
Food neophobia as a function of socio-political ideology moderated by implicit association between novel food and foreigner outgroups (Study 2). Note: Error bars represent standard errors.

In order to test the main hypotheses that conservatism increases food neophobia moderated by the associations between novel food and both foreigner outgroups and threat, we performed two linear regressions, one on each food neophobia measure. We entered as predictors the conservatism index, both BIAT scores, and the two interaction terms along with the covariates. The results are displayed in Table 4. First, in line with Study 1, ideology emerged as the only predictor (except for openness to experience, which also significantly predicted FNS-R score) to retain statistical significance after controlling for the others. Second, as expected, the effect of ideology on both food neophobia measures was moderated by the implicit association between novel food and outgroup. In other words, as depicted in Fig 2, food neophobia increased with higher levels of socio-political conservatism, but only for participants who more strongly associated novel food with foreigners. In contrast, although participants also tended to associate novel food with general threat, this association neither increased food neophobia nor moderated the ideology–food neophobia relation. Although people seem to intuitively and automatically feel threatened by novel food, we presume they consciously and explicitly know that these foods are not actually dangerous, at least in Western culture where food safety is taken for granted. Though this awareness does not prevent people from having an automatic threatened reaction, such a reaction nevertheless does not seem to explain food neophobia.

**Table 4:** Results of linear regression on participants’ food neophobia (both FNS-R and WTNF scores) predicted by covariates and socio-political ideology moderated by implicit associations between novel food and both foreigner outgroups and threat (Study 2).

	FNS-R						WTNF			
	β	*t*	*p*	95% CI		Β		*t*	*p*	95% CI
Constant		44.78	< .001	2.41, 2.64				51.65	< .001	.68, .73
General neophobia	.17	1.88	.063	-.01, .26		.06		.63	.530	-.02, .04
Openness to experience	**-.24**	**-2.93**	**.004**	**-.30, -.06**		.13		1.50	.137	-.01, .05
Disgust sensitivity	-.07	-.81	.418	-.20, .08		-.04		-.37	.713	-.04, .03
Sensation seeking	-.07	-.74	.464	-.21, 09		.18		1.71	.089	.00, .07
Ideology	**.31**	**3.85**	**< .001**	**.12, 37**		**-.29**		**-3.35**	**.001**	**-.08, -.02**
Novel food-Outgroup BIAT	**.21**	**2.66**	**.009**	.04, .28		-.10		-1.20	.232	-.05, .01
Novel food-Threat BIAT	.01	.06	.952	-.12, .12		.00		-.02	.986	-.03, .03
Ideology X Novel food-Outgroup BIAT	**.23**	**2.90**	**.004**	**.06, .32**		**-.18**		**-2.21**	**.029**	**-.07, .00**
Ideology X Novel food-Threat BIAT	-.03	-.34	.737	-.17, .12		.01		.11	.915	-.03, .04
	*R*^*2*^ = .33, *F*(9,121) = 6.51, *p* < .001, *f*^2^ = .48				*R*^*2*^ = .22, *F*(9,121) = 3.77, *p* < .001, *f*^2^ = .28					

Therefore, the results of Study 2 confirmed the link between socio-political conservatism and food neophobia and supported the social—but not existential—explanation of this relation. Drawing on previous research, we assumed in this study that conservative individuals would hold more negative attitudes toward foreigners [e.g., 26], and be more sensitive to threat [15]. To further test both explanations, we performed two experiments wherein we directly manipulated the aspects of conservatism that should interact with the novel food–threat association and the novel food–foreigners association, namely threat sensitivity and attitude toward foreigners. More importantly, the experimental method overcomes two potential problems with correlational studies: common method variance bias and inability to establish the causal direction of the ideology–food neophobia relation.

In addition, in the subsequent studies, we used a more behavioral measure of food neophobia (i.e., menu choice in a mock restaurant scenario) as a dependent variable in addition to FNS-R score. Finally, in Study 2, the participants were students, which may reduce the generalizability of the results. Therefore, we attempted to recruit older and more heterogeneous samples in the subsequent experiments.

## Study 3: An experimental test of the existential explanation

Drawing on Nail et al. [68], the present experimental study aimed to test whether threatening participants made the relation between conservatism and food neophobia decline or disappear. Indeed, if conservatives are more neophobic than liberals because they are more sensitive to threat, inducing threat in liberals should bring them closer to conservatives in terms of food neophobia. Alternatively, if only conservatives are affected by the manipulated threat due to their higher threat sensitivity, this could also strengthen the association between ideology and food neophobia. In any case, a significant interaction effect between ideology and manipulated threat on neophobia level would mean that threat plays a role in the ideology–neophobia link. Therefore, we compared a threat condition with two control conditions—a neutral/positive condition and a hassle condition—added to control for a possible effect of general negative mood on exploratory behavior. We chose a mortality salience manipulation unrelated to the food domain because reinforcing the novel food–threat association would make all participants more neophobic (in short, who wants to eat a potentially poisoned dish?) and violates the precondition of semantic autonomy between the explanans and the explanandum that genuine predictive models should guarantee [69].

### Method

#### Participants and procedure

A total of 530 Italian respondents, aged 18 to 84 years, were recruited through mailing lists and snowball sampling. Respondents filled out an online pre-questionnaire at T_1_ and left their email in order to receive the link to a second questionnaire containing the manipulation and dependent variables, approximately one week later (T_2_). The pre-questionnaire included socio-demographic information (age, gender, education, nationality, occupation, diet, allergies), the eight items assessing ideology already used in previous studies (α = .68), the FNS-R [45] (α = .87), and two measures of authoritarianism not relevant for the following analyses.

Of the initial participants, 300 (56.6%) completed the second questionnaire at T_2_. There were no significant differences between the two samples in terms of gender (χ^2^(1, *N* = 530) = .47, *p* = .491), education (*t*(528) = 1.19, *p* = .233), conservatism (*t*(528) = .07, *p* = .944), or food neophobia (*t*(528) = 1.22, *p* = .223). Instead, participants who completed the entire study were slightly but significantly younger (*M* = 29.93 years, *SD* = 12.76) than those who did not (*M* = 33.63 years, *SD* = 14.33; *t*(461.51) = 3.08, *p* = .002, *d* = .27).

At the beginning of the second questionnaire, participants were randomly assigned to conditions of two different experiments: Study 3 and another study not reported in this paper. The two samples did not differ in terms of gender, education, ideology, or food neophobia (all *p*-values > .145). Again, however, Study 3 participants were slightly but significantly younger (*M* = 28.42 years, *SD* = 11.29) than participants in the other study (*M* = 32.33 years, *SD* = 14.42; *t*(217.77) = 2.39, *p* = .018, *d* = .29). Yet, within such a wide age range (18–84 years), this difference was not dramatic.

Study 3 included 178 participants. Five were dropped from the analyses because of a large Cook’s distance [40] (one was in the control condition, one in the hassle condition, and three in the threat condition). The final sample consisted of 116 women and 57 men, aged 18 to 61 years (*M* = 28.47, *SD* = 11.30); 63.6% were students and 42.8% were employed. A sensitivity analysis conducted with G*Power [41], assuming an α of 0.05 and power of 0.95, showed that our sample was sufficient to detect small effects of *f*^2^ = .06 for a linear multiple regression with six predictors.

#### Experimental materials

In the second questionnaire, participants were asked to carefully read a short passage (143–152 words) and imagine themselves in the described situation. The first sentence introduced the scenario in the same way for all participants, asking them to “[i]magine being on a flight to reach Malurola, a tourist destination abroad, a 4,000 km distance from the town you live in” (Malurola is a fictitious place). The subsequent section varied according to the experimental condition (all manipulations are fully reported in S1 Appendix). A plane crash scenario (a thunderstorm with many frightening elements) was described in the threat condition (*N* = 57). The scenario ended with emotions of fear, and participants were not told how the situation turned out. Only when participants were asked to imagine going to the restaurant in Malurola did they learn that the danger had been averted. To make the manipulation more effective, the passage ended with a request to “describe in the following space the emotions that the thought of your death induces in you.” In the neutral control condition, a normal, comfortable flight was described instead (*N* = 59), ending with the protagonist starting a new book just purchased at the airport. Participants were then asked to “describe in the following space the emotions you feel when you start a new book.” Finally, in the hassle control condition (*N* = 57), the protagonist dealt with a trip full of hassles, queues, and delays, and the passage ended by asking participants to “describe the emotions you feel when you happen to waste time with queues and delays.”

After the manipulation, participants reported the extent to which they felt each of the following nine emotions (adapted from [70–73]): scared, upset, distressed, angry, annoyed, hassled, happy, enthusiastic, and relaxed. Responses were given on a 5-point scale ranging from *not at all* to *very much*. An exploratory factor analysis including these nine emotions revealed three factors explaining 74.41% of the variance (positive factor loadings > .77; “relaxed” loaded negatively, -.58, on the first factor). Therefore, three different emotion scores were computed: “threatened” as the mean of the scared, upset, distressed, and reverse-scored relaxed responses (α = .83); “annoyed” as the mean of the angry, annoyed, and hassled responses (α = .86); and “happy” as the mean of the happy and enthusiastic responses (α = .76, *r*(178) = .62, *p* < .001).

After this manipulation check, the dependent variable was assessed by asking participants to imagine being very hungry and going to an international restaurant located in Malurola. They read a menu including five courses and were asked to select only one dish out of six for each course. Each course included three familiar and three unfamiliar dishes, listed with their name and a brief description in parentheses (the full menu is reported in S2 Appendix). In other words, participants could choose either a familiar or an unfamiliar item for each course. As the courses were presented in their usual order according to the classic Italian meal structure (appetizers, first course, second course, side dish, and dessert), we did not consider the order in which participants selected familiar and novel food items. We computed a neophobic choice score as the count of familiar dishes selected from the menu (range: 0–5). Participants then completed the FNS-R [42] again (α = .88).

Some of the menu dishes were taken from the Study 2 WTNF test, and some were pre-tested in a pilot study. However, as we also used a few non-pre-tested food items, we included a familiarity check at the very end of the questionnaire, asking participants to indicate how familiar to them each menu dish was on a 5-point scale ranging *from not familiar at all* to *absolutely familiar*. A one-sample *t*-test confirmed that the means of the 15 supposedly familiar items were significantly higher and the mean of the 15 supposedly unfamiliar items were significantly lower than the midpoint of the scale (i.e., 3; all *p*-values < .001).

### Results and discussion

Table 5 presents the descriptive statistics and correlations for all variables. A multivariate analysis of variance (MANOVA) showed that participants assigned to different conditions were not different in terms of ideology, *F*(2,170) = 1.06, *p* = .348, η^2^ = .01, or food neophobia, *F*(2,170) = .66, *p* = .516, η^2^ = .01, measured at T_1_. Gender and education level affected neither dependent nor independent variables. Since age was positively correlated with conservatism (*r*(173) = .23, *p* = .002), we controlled for it in the subsequent models.

**Table 5:** Descriptives and correlations for Study 3 measures.

	*M*	*SD*	2	3	4	5	6	7
1. Ideology	2.22	.65	.29***	-.02	-.07	-.03	.29***	.27***
2. FNS-R at T_1_	2.24	.94	-	.06	.07	-.04	.45***	.88***
3. Threatened	2.22	.92		-	.48***	-.46***	.09	.05
4. Annoyed	1.72	.94			-	-.31***	.09	.09
5. Happy	2.66	.94				-	.01	-.06
6. Neophobic choice	2.53	1.81					-	.55***
7. FNS-R at T_2_	2.29	.91						-

*** *p* < .001.

As a manipulation check, we performed the same MANOVA on the emotions scores and found that our mortality salience manipulation (*M* = 2.74, *SD* = .99) induced feelings of threat more than both control conditions (neutral condition: *M* = 1.88, *SD* = .62; hassle condition: *M* = 2.06, *SD* = .90; Bonferroni post-hoc comparison *p-*values < .001), *F*(2,170) = 16.54, *p* < .001, η^2^ = .16. A regression analyses ran on each emotion score, entering two dummy variables for the experimental conditions (threat = 1, hassle = 1), the ideology score and its interaction with both dummies, showed that the main effect of threat condition on feelings of threat was not moderated by participants’ conservatism, β = -.09, *t*(167) = -.84, *p* = .348. Participants in the threat condition were also less happy (*M* = 2.33, *SD* = 1.05) than those in both control conditions (neutral condition: *M* = 2.94, *SD* = .71; hassle condition: *M* = 2.70, *SD* = .95; Bonferroni post-hoc comparisons *p* = .001 and *p* = .097, respectively; *F*(2,170) = 6.52, *p* = .002, η^2^ = .07). The regression analyses showed that this main effect of threat condition on happiness, β = -.28, *t*(167) = -3.23, *p* = .001, was stronger at lower levels of conservatism β = .22, *t*(171) = 2.18, *p* = .030. In contrast, no significant differences emerged for annoyance emotions, *F*(2,170) = 1.11, *p* = .333, η^2^ = .01.

The same MANOVA was performed on the dependent variables, showing that our manipulation did not affect either neophobic choice, *F*(2,169) = .76, *p* = .467, η^2^ = .01, or FNS-R scores at T_2_, *F*(2,169) = .46, *p* = .630, η^2^ = .00. However, we expected that, when threatened, liberals would become as neophobic as conservatives or, alternatively, that conservatives would become even more neophobic than liberals if the manipulation only affected them. As the manipulation check showed that liberals and conservatives were equally threatened in the threat condition, we lean toward the first hypothesis. In any case, both hypotheses would result in an interaction between ideology score and threat manipulation after controlling for the baseline level of food neophobia. This interaction would support the existential explanation of the conservatism–food neophobia relation. To test this hypothesis, we ran a hierarchical linear regression on the neophobic choice score, entering age, conservatism, and FNS-R score measured at T_1_ as first-step predictors and two dummy variables for the experimental conditions (threat = 1, hassle = 1) as well as the ideology by threat interaction term at the second step. The results confirmed that ideology significantly predicted participants’ neophobic choice, β = .23, *t*(169) = 3.24, *p* = .001, even after controlling for FNS-R score at T_1_, β = .40, *t*(169) = 5.87, *p* < .001, and age, β = -.21, *t*(169) = 3.11, *p* = .002 (first-step *R*^*2*^ = .27, *F*(3,169) = 21.34, *p* < .001, *f*^2^ = .38). In contrast, none of the predictors included at the second step affected the dependent variable (Δ*R*^2^ = .02, *F*(3,166) = 1.27, *p* < .287, *f*^2^ = .02). In other words, our threat manipulation did not affect participants’ food neophobia levels, either alone or in interaction with ideology.

We must acknowledge that, although participants in the threat condition felt significantly more threatened and less happy than those in the two control conditions, the average level of threat emotions was lower than the midpoint even in the former group, possibly suggesting that our manipulation did not completely succeed. Nonetheless, the same regression analyses performed after removing participants with a threatened score ≤ 3 produced the same findings reported above (*N* = 139).

These results, in line with Study 2, disconfirm the existential explanation of the ideology–food neophobia relation, suggesting that conservative individuals are not more food neophobic than liberals because they are more sensitive to threat (or, at least, that such an effect may be so small our sample size did not allow to detect it). In addition, the manipulated threat did not increase food neophobia. This is consistent with Study 2, wherein the threatening nature of novel food did not affect participants’ food neophobia levels or moderate the ideology–food neophobia relation, suggesting that nowadays—at least in developed countries, where most consumers are not seriously or consciously concerned about food safety—danger may be no longer a relevant motivation for refusing novel food.

Study 3 also showed—again, in line with studies 1 and 2—that conservative individuals are more neophobic than liberals. This also emerged using a more behavioral (although set in a fictitious scenario) measure of food neophobia—namely, a meal choice task—and persisted even when controlling for trait food neophobia assessed at T_1_.

## Study 4: An experimental test of the social explanation

In the present experiment, we intended to re-test the social explanation of the ideology–food neophobia relation already yielded in Study 2. To this end, we devised a 2-condition between-participants experimental design manipulating the other aspect of socio-political conservatism that, according to our initial hypotheses, should explain why conservatives are more neophobic than liberals (namely, attitude toward foreigner outgroups). Specifically, we hypothesized that inducing a positive (vs. negative) attitude toward an outgroup and presenting novel foods associated with that outgroup would make the relation between ideology and food neophobia decline or disappear (see [68]). Indeed, if conservatives are more neophobic than liberals because they hold more negative attitudes toward foreigners, whom they associate with novel food, inducing a positive attitude in conservatives should bring them closer to liberals in terms of food neophobia (i.e., make them less neophobic). Alternatively, inducing a negative attitude toward foreigners in liberals could make them more neophobic, like conservatives. In both cases, manipulating this attitude should reduce or eliminate the association between ideology and food neophobia. We also assessed participants’ emotions to rule out the possibility that positive mood, rather than attitude toward the outgroup, could make individuals more explorative and thus affect the dependent variables.

### Method

#### Participants

A total of 187 participants recruited through mailing lists and snowball sampling filled out an online pre-questionnaire at T_1_ and provided their emails so that they could receive the link to a second questionnaire approximately one week later (T_2_). Of these initial participants, 127 (67.9%) completed the second questionnaire at T_2_. There were no significant differences between the two samples in terms of gender, χ^2^ (1, *N* = 187) = .20, *p* = .656, age, *t*(185) = 1.47, *p* = .143, 95% CI [-4.83, .71], education, *t*(94.42) = 1.78, *p* = .078, 95% CI [-.09, 1.65], ideology, *t*(185) = 1.47, *p* = .143, 95% CI [-.30, .04] and food neophobia, *t*(185) = 1.21, *p* = .226, 95% CI [-.43, .10]. Five participants were dropped because of a large Cook’s distance [54] (two were in the negative and three in the positive attitude condition). The final sample consisted of 105 women and 17 men, aged 20 to 61 years (*M* = 30.10, *SD* = 9.00); 44.3% were students and 50.0% were employees. A sensitivity analysis conducted with G*Power [41], assuming an α of 0.05 and a power of 0.95, showed that our sample was sufficient to detect small effects of *f*^2^ = .09 for a linear multiple regression with five predictors.

#### Procedure and materials

In the first questionnaire, participants provided socio-demographic information and completed the measure of socio-political ideology (α = .68), the FNS-R (α = .87), and two measures of authoritarianism not relevant for the following analyses. In the second questionnaire, participants were asked to carefully read a short passage (221–288 words) and imagine themselves in the described situation. The first sentence introduced the scenario and was the same for all participants: “Imagine you are on holiday in Malurola, a tourist destination abroad, a 4,000 km distance from the town you live in.” We chose to have participants meet the target outgroup in the latter’s home country because the opposite scenario (i.e., immigrants coming to participants’ home country) could result in perceived threat and thus confound our manipulation, which needed to affect attitude only. The subsequent part of the passage varied by experimental condition (all manipulations are fully reported in S3 Appendix). In the negative attitude condition (*N* = 62), Malurola inhabitants were described as very cold, rude, and hostile (although we ensured we did not depict them as threatening), whereas in the positive attitude condition (*N* = 60) they were described as particularly kind and friendly.

We then assessed, in randomized order, participants’ emotions and attitudes toward Malurola inhabitants as a manipulation check. On a 5-point scale ranging from *not at all* to *very much*, participants reported how angry, alarmed, sad, disgusted, glad, happy, delighted, and satisfied they felt (adapted from [72]). An exploratory factor analysis revealed a single factor explaining 70.56% of variance. Therefore, after rescaling negative emotions, we computed a single positive emotions score (α = .94).

Attitude toward the outgroup was measured using two items: Participants reported their global impressions of Malurola inhabitants on a 5-point scale ranging from *completely negative* to *completely positive* and indicated how much they liked them on a 5-point scale ranging from *not at all* to *very much*. We then computed a single attitude score (α = .97, *r*(122) = .94, *p* < .001).

Next, as in Study 3, the fictitious restaurant scenario was introduced. However, in Study 4, we forced and assessed the association between novel food and the specific outgroup toward which the attitude was manipulated. To this end, we stated that both Italian and typical Malurolian dishes could be found on the menu: Before choosing their meal, participants needed to indicate the culture of origin of each food listed. Some participants failed to correctly associate each food with its country and, given the importance of the food–group association, these participants were dropped from the main analyses: 84.4% of participants were retained in the final sample, as they correctly recognized at least 28 out of 30 familiar foods as Italian and novel foods as Malurolian. They were equally distributed among conditions, χ^2^ = .68 (1, *N* = 122), *p* = .408, and the number of correct associations did not correlate with either conservatism or FNS-R score. Based on the subsequent meal choice task, a neophobic choice score was computed as in the previous experiment. Finally, participants filled out the FNS-R [45] (α = .87).

### Results and discussion

Table 6 displays descriptive statistics and correlations for all measures. Gender, age, and education level did not affect either dependent or independent variables. Further, there were no differences in participants’ levels of food neophobia and ideology measured at T_1_ as a function of the experimental condition, *p* > .07.

**Table 6:** Descriptives and correlations for Study 4 measures.

	*M*	*SD*	2	3	4	5	6
1. Ideology	1.87	.57	.34***	-.21*	-.23*	.35***	.38***
2. FNS-R at T_1_	1.91	.84	-	-.17	-.13	.57***	.87***
3. Positive emotions	3.24	1.13		-	.82***	-.29**	-.20*
4. Attitude	2.97	1.51			-	-.37***	-.18
5. Neophobic choice	1.75	1.65				-	.60***
6. FNS-R at T_2_	2.00	.85					-

* *p* < .05

** *p* < .01

*** *p* < .001.

To test whether our manipulation affected manipulation check measures and dependent variables, we ran an independent samples t-test. The results showed a significant effect of condition on emotions, *t*(120) = 13.63, *p* < .001, *d* = 2.47, 95% CI [1.47, 1.97], attitude, *t*(120) = 22.56, *p* < .001, *d* = 4.09, 95% CI [2.45, 2.92] and neophobic choice, *t*(120) = -3.18, *p* = .002, *d* = -.58, 95% CI [-.34, -1.47], but not on FNS-R score assessed at T2, *t*(119) = -.37, *p* = .713, *d* = .07, 95% CI [-.36, .25]. Means and standard deviations are reported in Table 7.

**Table 7:** Means and standard deviations as a function of experimental conditions (Study 4).

	Negative attitude condition		Positive attitude condition	
	*M*	*SD*	*M*	*SD*
Positive emotions***	2.41	.76	4.13	.63
Attitude***	1.68	.67	4.37	.64
Neophobic choice**	2.29	1.77	1.38	1.34
FNS-R at T2	2.01	.86	1.95	.82

** Means on this row are significantly different with *p* < .01.

*** Means on this row are significantly different with *p* < .001.

**Fig 3:**
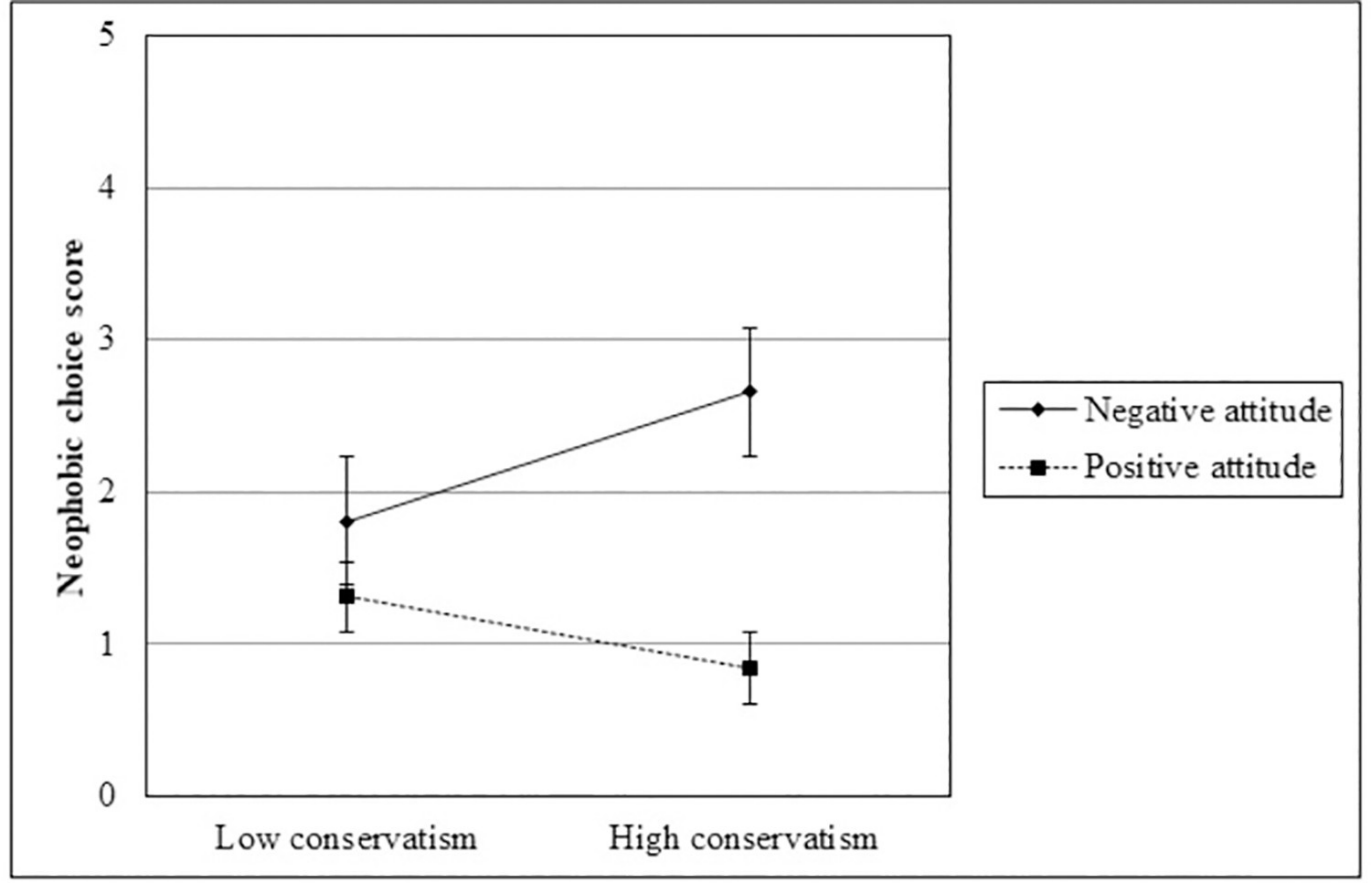
Neophobic choices as a function of socio-political ideology moderated by the experimental condition (Study 4). Note: Error bars represent standard errors.

To test the main hypothesis that inducing a negative (vs. positive) attitude toward Malurolians could eliminate or reduce the association between ideology and food neophobia, we performed a linear regression analysis on the neophobic choice score, entering as predictors conservatism level, dummy-coded experimental condition (1 = positive attitude), and their interaction term and also controlling for FNS-R score assessed at T_1_ and positive emotions. The results are reported in Table 8 and showed that, even after controlling for FNS-R score at T_1_ and positive emotions, the conservatism measure and the experimental manipulation significantly predicted participants’ neophobic choice. More importantly, the ideology by condition interaction was also significant: As portrayed in Fig 3, more conservative participants made more neophobic choices in the negative attitude condition, *B* = .42, *SE* = .16, *p* = .010, 95% CI [.10, .74], whereas the ideology–neophobia relation was not significant in the positive attitude condition, *B* = -.23, *SE* = 21, *p* = .271, 95% CI [-.65, .18]. In other words, as expected, the positive attitude toward a foreigner outgroup associated with novel food weakens conservatives’ proneness to food neophobia, bringing them closer to liberals on this dimension.

**Table 8:** Results of linear regression on participants’ neophobic choice (Study 4).

	β	*t*	*p*	95% CI
(Costante)		10.25	< .001	1.80, 2.66
FNS-R at T_1_	**.54**	**6.78**	**< .001**	**.64, 1.17**
Positive emotions	.07	.63	.527	-.26, .50
Exp.condition (1 = positive attitude)	**-.35**	**-3.03**	**.003**	**-1.91, -.40**
Ideology	**.26**	**2.63**	**.010**	**.10, .74**
Ideology X Condition	**-.24**	**-2.54**	**.013**	**-1.16, -.14**
	*R*^2^ = .47, *F*(5,97) = 17.46, *p* < .001, *f*^2^ = .90			

Finally, we wanted to explore whether this interaction effect also extended to trait food neophobia and thus performed a further analysis using PROCESS, the SPSS macro provided by Hayes [74]. We tested model 8, setting 5,000 bootstrap resamples, to estimate the effect of ideology on FNS-R score at T_2_ both directly and indirectly through neophobic choice score, with both direct and indirect effects moderated by experimental condition. We controlled for positive emotions and FNS-R score at T_1_ in the mediator model. In addition to the previously reported results on neophobic choice, this analysis showed that, although only neophobic choice significantly and directly affected FNS-R scores at T_2_, β = .59, *p* < .001, 95% CI [.35, .65], ideology had a conditionally indirect effect through neophobic choice, *B* = -.20, *SE* = .08, 95% CI [-.40, -.06], increasing FNS-R scores at T_2_ in the negative attitude condition, *B* = .13, *SE* = .06, 95% CI [.03, .26], but not in the positive attitude condition, *B* = -.07, *SE* = .05, 95% CI [-.18, .02]. This suggests that our manipulation, which contingently improved participants’ attitudes toward a specific outgroup associated with the presented novel food, was effective in rendering conservatives similar to liberals in terms of intergroup attitudes, thus dissolving their difference also in terms of food neophobia, not only as assessed by a mock meal choice task but even (although indirectly) via the trait measure.

Overall, these results, as well as confirming that conservative individuals are more neophobic than liberals with respect to their food choice, supported the social explanation of the ideology–food neophobia link, in line with Study 2. This suggests that food neophobia has social roots originating from the association between novel foods and foreigner outgroups, who are usually disliked by conservative individuals.

## General discussion

Despite the well-known social meanings of food and the common ground of resistance to change that food neophobia shares with socio-political conservatism [17], this is the first empirical investigation of the relation between ideology and food neophobia. We conducted four studies with different samples (university students and more heterogeneous adult participants) and various measures of the dependent variable (trait measure, i.e., FNS-R in all studies; a willingness to taste-test novel food, including different items in Studies 1 and 2; and a fictitious meal choice task set in a mock restaurant in Studies 3 and 4). Across all studies, we showed that conservative ideology is associated with food neophobia (*r*(627) = .32, *p* < .001).

Given the many common antecedents of the two constructs, one could expect that this association might be spurious. Instead, we found evidence of a direct and robust link that remained significant even after controlling for those common antecedents (Studies 1 and 2) and for trait food neophobia assessed one week prior to completing the fictitious meal choice task (Studies 3 and 4). In addition, socio-political ideology outperformed all other food neophobia predictors, including general neophobia, openness to experience, disgust sensitivity, sensation seeking, and education level.

Furthermore, the present contribution explored possible explanations for the ideology–food neophobia relation, testing two non-alternative hypotheses. The first, which we labeled the existential explanation, relies on the danger category of Rozin and Fallon’s [12,13] food rejection taxonomy, combined with conservatives’ high threat sensitivity. Study 2 showed that novel foods were actually associated with general threat but this implicit association did not predict food neophobia, either alone or as a moderator of the ideology effect. Consistently, in Study 3, the experimentally manipulated threat did not affect food neophobia, either alone or as a moderator of the ideology effect. These findings, obtained across different samples (students in Study 2 and adults “from the street” in Study 3) and methods (correlational, using implicit measures in Study 2, and experimental in Study 3), led us to reject the existential explanation, suggesting that conservatives are not more neophobic than liberals because they are more sensitive to threat. In addition, these results might lead us to infer that, although food neophobia evolutionarily developed to protect eaters in potentially dangerous environments, the general category of danger may no longer be as relevant a motivation for refusing novel food, at least in Western cultures where food safety is not a serious concern for consumers [75]. Despite this consistency, it may be premature to conclude that the threatening nature of novel food is uninfluential in adults’ food neophobia. Indeed, in our procedures, the use of general threat stimuli (in Study 2) and a general mortality salience scenario (in Study 3) does not allow to exclude the possibility that a more specific food-related threat might affect food neophobia. Instead, the choice to focus on general threat seems suitable for testing whether (and disproving that) conservatives are more neophobic than liberals because they are more threat sensitive. However, further studies using different measures and manipulations of threat are needed to support or oppose our preliminary interpretation.

Our second hypothesis, which we called the social explanation, relies on the disgust category of the same food rejection taxonomy [12,13]—more specifically, on the cognitive association between novel food and social minorities, combined with conservatives’ negative attitudes toward such outgroups. Study 2 showed that novel foods were associated with foreigners and that this implicit association significantly predicted food neophobia, both alone (but only for the trait measure) and, more importantly, as a moderator of the ideology effect. In other words, conservatism appeared to significantly increase food neophobia when participants associated novel food with foreigners. In line with these findings, Study 4 showed that experimentally making conservatives’ intergroup attitudes more positive—and thus more similar to liberals’—overrode the ideology–food neophobia link when the target outgroup was associated with the proposed novel dishes. The present results confirmed the social explanation of the ideology–food neophobia relation: Conservatives are more reluctant than liberals to eat unfamiliar foods because they hold more negative attitudes toward social minorities, who are in large part responsible for the evolution and diversity of the modern food supply.

We must acknowledge some limitations of the present work. For one thing, our samples are not representative of the Western population. First, there were many more women than men among our participants. However, this should not be a problem, as both the literature and the present work did not find gender differences in our crucial (dependent and independent) variables: Gender did not affect either conservatism or any of the food neophobia measures in any of our studies or across them (*p* > .27). In addition, across the four studies, we checked for and found the same correlations between socio-political ideology and food neophobia in both male, *r*(138) = .29, *p* < .001, and female participants *r*(489) = .33, *p* < .001, *z* = .41, *p* = .34. Second, despite our efforts to recruit a heterogeneous sample, it eventually became unbalanced also in terms of socio-political orientation: The average levels of conservatism and food neophobia were indeed low across all our studies. However, we believe that, if differences emerged when comparing liberals with only mildly conservative individuals, these differences should even strengthen if a larger amount of more conservative participants were included in our samples. Third, our participants were all Italian: Although we supposed that the examined processes would apply to other Western societies, further studies conducted in different countries and with more heterogeneous samples are advisable to verify the generalizability of our findings.

In addition, although the social explanation would concern social minorities in general, as long as they are associated with novel food, we assessed the association between novel food and low-status foreigners (in Study 2) and manipulated attitudes toward a specific fictitious foreigner outgroup (in Study 4). For these reasons, the present research focused on exotic or ethnic novel food. However, not all outgroups are equivalent and (dis)liked in the same way (e.g., [76]), and we formulated the social explanation hypothesis considering that most novel food is associated with low-status or minority outgroups that are particularly disliked by conservatives, such as immigrants. Therefore, we must be cautious in generalizing our conclusions, and further research is needed to extend our results to other minority and derogated outgroups, such as vegans, and non-ethnic novel food, such as vegan food. Another drawback is that we did not assess perceived realism of the scenarios used in the experimental studies. Moreover, as this is a first investigation into the ideology–food neophobia link, we strove to keep separate the two alternative, but not mutually exclusive, explanations. However, in real life, such explanations often co-occur, as in the case of intergroup threat, and more work is needed to explore this natural co-variation.

Finally, an alternative explanation of our findings could be that conservatives often live in less culturally diverse areas than liberals and thus have less opportunity to be exposed to novel food, which is a known means of reducing food neophobia [77–79]. Unfortunately, although we paid attention to a number of covariates that could have made the ideology–food neophobia relation spurious, we failed to control for residential area. However, other data we collected for different purposes ([80]) can help rule out this alternative explanation in terms of familiarity. A convenience sample of adults living in cities or towns that differ in dimension and level of cultural or ethnic heterogeneity was asked to estimate the number of ethnic restaurants in both their town and their neighborhood. Ideology still predicted food neophobia in a regression controlling for both perceptions, and this held true also when the other covariates (general neophobia, openness to experience, disgust, education) were included in the model. This suggests that the ideology–food neophobia relation is not spuriously due to differences in conservatives’ and liberals’ living area homogeneity, which would also affect exposure to novel food, and thus reinforces our social explanation.

Notwithstanding these limitations, the present work has the strength of combining different and apparently unrelated literatures, with theoretical and practical implications for both. Previous research on the antecedents of food neophobia has focused exclusively on structural factors, personality, and emotions (for a review, see [11]). Although modeling, indicating that eaters imitate the neophilic or neophobic behavior of significant others [81], has been considered among the intervention strategies, and parent–child resemblance in food neophobia has been investigated in some studies [6,82,83], a social attitude account of food neophobia has not yet been advanced.

Associating a food category with an ingroup with which one identifies can improve one’s attitudes toward those foods and promote their consumption [84]. Hence, it is not surprising that associating a food category with a disliked outgroup can induce negative attitudes and avoidance reactions toward those foods. These four studies introduced socio-political ideology as a new causal antecedent of food neophobia, found evidence that it is actually the most powerful among other predictors, and confirmed a social explanation for this relation. This contributes to framing food neophobia within a network of correlates that are at various levels of analysis (i.e., intrapersonal, interpersonal, intergroup, and cultural). Therefore, we believe that our results can deeply change the way in which the food neophobia issue is addressed, moving the interest from individual causes and solutions to a more far-reaching understanding of it.

This wider perspective has important practical implications and generates new research questions. Study 4 indicated that conservatism increased food neophobia only when intergroup attitudes were negative. Put another way, when novel foods and outgroups were associated in individuals’ memory, improving intergroup attitudes reduced food neophobia, at least for moderates (mean score; *B* = -1.16, *SE* = .38, *p* = .003) and conservatives (+1 SD; *B* = -1.82, *SE* = .45, *p* < .001)—that is, those who most need both improvements. Instead, our results suggest that the thus far identified intervention strategies of food neophobia reduction, such as repeated exposure to novel food, may be not effective for conservatives who associate novel food with disliked outgroups. Therefore, future studies should explore different ways to foster eaters’ curiosity and enlarge their food repertoires, also fitted to the target’s socio-political orientation and social attitudes. For instance, to reduce people’s food neophobia, it should be effective to induce associations between novel foods and positive outgroups, between novel foods and ingroups, or—more difficult in real contexts but doubly useful—to improve the attitude toward the outgroups associated with those novel foods.

Concerning the ideology literature, the present work is framed in the model of political ideology as motivated social cognition [15–17]. We believe that it may offer an important contribution by confirming that the differences between conservatives and liberals, which are deeply rooted in their epistemic, existential, and relational needs, also extend to food attitudes and choices [19–23]. An even more relevant contribution of the present research is related to intergroup contact theory [85,86]. Although we could not find studies investigating this possibility, sharing food has always been considered a means of socializing and consolidating friendships [87] and thus can be an effective special case of intergroup contact. Indeed, the idea of improving intergroup relations through “multi-ethnic dinners” seems popular in small communities across the world, and some research suggests that sharing food is interpreted as a sign of social intimacy [88], while eating the same food leads to increased trust and cooperation among strangers [89]. Our results indicate that improving intergroup attitudes, in addition to being inherently beneficial, can also help enlarge individuals’ array of accepted food, but do not exclude that the reverse may be true—that is, it is possible that increasing individuals’ acceptance of novel food could help improve their intergroup attitudes. In fact, we can speculate that the ideology–food neophobia relationship is circular, with conservative orientation reinforcing the tendency to refuse novel food, which in turn could foster conservatism by worsening attitudes toward the associated outgroups. Further research is needed to explore all of these issues in greater depth.

## Supporting information

S1 Appendix Study 3 manipulations. (PDF)

S2 Appendix Studies 3–4 restaurant menu. (PDF)

S3 Appendix Study 4 manipulations. (PDF)
