# Supplementary material for: Dining with liberals and conservatives: The social underpinnings of food neophobia
Source: PLoS One. 2022 Jan 27;17(1):e0262676. doi: 10.1371/journal.pone.0262676 (PMC8794126; doi:10.1371/journal.pone.0262676)
Supplement: S1 Appendix — (PDF) [file pone.0262676.s001.pdf]

# S1 Appendix – Study 3 manipulations

Imagine being on a flight to reach Malurola, a tourist destination abroad, a 4000 km distance from the town you live in.

THREAT CONDITION

An hour after take-off, the airplane is in the grips of a thunderstorm causing very strong turbulence. Passengers are invited to remain in their seats and fasten their seatbelts. The pilot decides to fly fast to get out the storm and this provokes a sudden air imbalance. The lights go out and you feel the oxygen is lacking. Your mask comes down immediately, but you are very nervous and it takes you some time to correctly wear it. You hear the other passengers yelling, everybody is terrified because of what is happening. There is full-scale panic, children are crying loudly and despairing and even the cabin crew appears really scared. You think that the airplane is falling and that you are about to die.

Describe in the following space the emotions that the thought of your death induces in you".

HASSLE CONDITION

When you arrive at the airport, you soon notice that it is particularly crowded. You look at the departure board and see that your flight is an hour late. You go to the check-in. There is a really long queue and people are crowded in front of the check-in desk. After three hours of waiting, you finally can embark your luggage, but you have to pay a surcharge because it weighs one pound more than it should. You proceed to the checks. There are many people, so also the checks and preboarding progress very slowly. On the airplane, the person sitting next to you borders your armrest and, after a few minutes, he begins snoring loudly. There is also some turbulence, so the flight arrives with an additional hour of delay.

Describe in the following space the emotions you feel when you happen to waste time with queues and delays.

CONTROL CONDITION

When you arrive at the airport, you look at the departure board and go to the check in. On your way toward boarding, you meet an acquaintance whom you have not met for a long time. You stop to greet and ask each other for the news of your lives. The flight is on time. There are not that many people, so in a short period you are on the airplane. You have a window seat and the chair is pretty comfortable. The weather is good, so the take-off and the flight go smoothly. You listen to some music and have a chat with the persons sitting next to you who already went on holiday in Malurola, and they offer you some useful advice. You look at the clouds outside the window and are also able to have a nap. You then decide to start reading the new book you have just bought at the airport.

Describe in the following space the emotions you feel when you start a new book.
