# Supplementary material for: Dining with liberals and conservatives: The social underpinnings of food neophobia
Source: PLoS One. 2022 Jan 27;17(1):e0262676. doi: 10.1371/journal.pone.0262676 (PMC8794126; doi:10.1371/journal.pone.0262676)
Supplement: S2 Appendix — (PDF) [file pone.0262676.s002.pdf]

S2 Appendix – Studies 3-4 restaurant menu

1) Antipasti
Tagliere di affettati italiani (prosciutto crudo e cotto, salame, coppa)*
Bruschette sicilian style (pane tostato con pomodorini e basilico)*
Insalata di mare (calamari, gamberetti, granchio, polpo)*
Uova centenarie (uova lasciate 100 giorni in acqua, sale, carbone e ossido di calcio)
Dolmaedes (involtini di riso avvolti nelle foglie di vite)
Souskai De Platano (insalata di platani con peperoncino, aglio ed olio)

2) Primi
Fettucine Alfredo (con burro e parmigiano)*
Spaghetti alla bolognese (con ragù di carne)*
Risotto alla boscaiola (con funghi misti, panna e formaggio)*
Manti (ravioli ripieni di agnello, conditi con yogurt all'aglio e burro al peperoncino)
Turtle soup (zuppa di carne di tartaruga, spinaci e uova)
Ramen (noodles con salsa di soia, verdure, funghi shiitake e sesamo nero) - replaced in Study 4 by Bokkeumbap (riso saltato con daikon fermentato, alghe jim, funghi shiitake e sesamo nero)

3) Secondi
Saltimbocca alla romana (involtini di vitello, prosciutto crudo e salvia)*
Baccalà alla veneziana (merluzzo mantecato con latte e cipolla)*
Polpette vegetariane alla parmigiana (polpette di melanzane con pomodoro e grana)*
Rollè di tempeh esotico (con ananas e papaya)
Camel Burger (hamburger di cammello servito con cipolle caramellate)
Kæstur hákarl (carne di squalo servita con panna acida, patate e cipolla)

4) Contorni
Insalata mista (lattuga gentile, pomodori e carote)*
Fantasia di asparagi (asparagi stufati al burro con formaggio filante)*
Contorno primavera (carote, fagiolini e piselli stufati con erba cipollina)*
Spiedini di topinambur e zucca con salsa di lattuga
Pak choy (cavolo cinese saltato con zenzero e peperoncino)
Babaganoush (caviale di melanzane con tahin, aglio e menta)

5) Dolci
Tiramisù (semifreddo con savoiardi, caffè e formaggio fresco)*
Crostata Luisa (crostata di pasta frolla con marmellata di albicocche*
Torta nera della nonna (torta soffice con pere e cioccolato)*
Manju (dolcetti di riso e grano saraceno ripieni di crema dolce di azuki)
Halva (dolce a base di semolino e uvetta, profumato al cardamomo)
Mjuk rabarberkaka (torta soffice al rabarbaro con scaglie di cocco)

*Familiar dishes.
