# Supplementary material for: Dining with liberals and conservatives: The social underpinnings of food neophobia
Source: PLoS One. 2022 Jan 27;17(1):e0262676. doi: 10.1371/journal.pone.0262676 (PMC8794126; doi:10.1371/journal.pone.0262676)
Supplement: S3 Appendix — (PDF) [file pone.0262676.s003.pdf]

**S3 Appendix – Study 4 manipulations**

Imagine being on holiday in Malurola, a tourist destination abroad at a 4000 km distance from the town you live in.

NEGATIVE ATTITUDE CONDITION

Since you arrived at the airport, you find a very hostile and cold atmosphere. Although the hotel told you there was a free shuttle service, you do not find anyone welcoming you at the airport and with great difficulty you finally hail a taxi that brings you to the hotel you booked. The driver does not speak your language and also looks unkind.  Once arriving at the hotel, he neither offers to help with your luggage and drops you off several feet further than necessary from the entrance.

As soon as you arrive at the hotel, you immediately notice that the staff is very cold and unfriendly and there is no pleasant welcome of any kind. After some rest, you decide to go out for a first visit to the new town and ask for guidance at the reception. You are surprised by the staff's bad manners and the little information they give you.You visit the town on foot for the first time and you meet people seemingly gloomy and rude, nobody says hello and everyone seems too busy going about their own business, uncaring of others. The inhabitants seem very cold and unfriendly, not only to each other but also towards tourists.

Some days after your arrival, you have not still been able to meet nor to talk with any native citizen: they are all very closed-off and almost seem annoyed by the presence of tourists. Most inhabitants have proven to be nasty, surly and not willing to show the beauty of the place. Nobody has given you any information and you have always had to make do with maps because as soon as by-standers on the street learn you are a tourist, they try to avoid you.

POSITIVE ATTITUTE CONDITION

Since you arrived at the airport, you immediately find a particularly warm atmosphere and meet a person welcoming you and offering to bring your luggage to the free shuttle, which drives you to the hotel you booked.

As soon as you arrive at the hotel, you are offered a welcome cocktail and accommodated in your room so you can freshen up and relax after the long journey. After some rest, you decide to go out for a first visit to the new town and ask for guidance at the reception. You are surprised by the staff's kindness and by the amount of useful information they give you.

You visit the town on foot for the first time and meet very friendly people. They all look very nice and happy, everyone says hello and seem particularly kind, not only to each other but also tourists.

Some days after your arrival, you have met many native citizens. Most of them have proven to be affable, helpful and willing to show you the beauty of the place. You have also been impressed by the kindness and politeness of the by-standers you asked for information from - all are very helpful and open towards tourists.
